# Incidence and concomitant factors of cesarean sections in the bitch: A questionnaire study

**DOI:** 10.3389/fvets.2022.934273

**Published:** 2022-09-02

**Authors:** Magdalena Schrank, Barbara Contiero, Antonio Mollo

**Affiliations:** Department of Animal Medicine, Production and Health, University of Padua, Legnaro, Italy

**Keywords:** cesarean section, dystocia, dog, stillbirth, parity

## Abstract

Dystocia in the canine species is a common problem, and elective cesarean sections (C-sections) have become more frequent in breeds that are at risk. The aim of this study was to evaluate the incidence of C-section and contributing factors and to compare data on elective and emergency C-sections (e.g., regarding stillbirth). Using a questionnaire, a total of 423 bitches of 80 breeds and their 899 litters were included. The mean number of litters per bitch was 2.1 ± 1.1 litters. The overall rate of stillbirth was 6.7%. Of all litters, 194 were born *via* C-sections (21.6%), of which 35 were declared as elective and 159 as emergency due to dystocia. Significantly more C-sections were performed in either small litters (1-2 pups) or large litters (>12 pups) (p < 0.001). Bitches that have had prior C-sections had a 4-fold increase in the risk of successive C-sections (RR = 4.54 (95%CI 2.56–7.70; *p* < 0.001). Furthermore, primiparous bitches of advanced age had a significantly higher incidence of emergency C-sections (*p* = 0.004). Stillbirth was significantly higher in emergency C-sections compared with that in elective C-sections (*p* = 0.003). Also, timing of intervention had a significant impact on stillbirth in emergency C-sections (*p* = 0.025). Within a breed-specific evaluation, significant differences were observed between breeds regarding incidence of C-section and stillbirth. Lesser-known breeds were represented in the population, and the results showed that the Norwich Terrier had the highest (51.6%) and the Gordon Setter had the lowest (4.8%) incidence of C-section (*p* < 0.001). The inclusion and evaluation of lesser-known breeds regarding incidence of C-section is of importance as it shows that certain breeds without phenotypical traits such as brachycephaly may also have an increased incidence of emergency C-section and stillbirth. We further conclude that more importance may be given to the age at first parturition concerning the occurrence of dystocia and the decision making regarding possible elective C-sections.

## Introduction

Dystocia in the dog is a common problem (1–3), and several risk factors have been described in mixed-breed as well as purebred canine populations (1–5). The medical treatment of dystocia is possible in certain cases, yet the success rate is often low for most cases requiring a cesarean section (C-section) (4, 6). Although certain risk factors for dystocia are well described, such as breed (7) or singleton pregnancy (8), others are often spoken about within the community of breeders (e.g., the size of the male in relation to the size of the female, prior C-sections of the female and others). Dystocia may have many causes and may in some cases be difficult to identify, especially by unexperienced breeders. It is important to anticipate which pregnancies are at particular risk in order to organize proper medical intervention to ensure the wellbeing of both the mother and her progeny. Elective C-sections are planned ahead of time and may be performed on either a “parturient” (with an open cervix) or “preparturient” (with a closed cervix) bitch (9). Most commonly the term elective C-section refers to a parturient C-section (9) in the absence of evident contractions, expulsion, and/or signs indicating dystocia. This approach has become more popular in small animal reproduction medicine over the last few years (9), particularly in bitches, which are considered to be at high risk of dystocia (8, 9). Various publications evaluated protocols and methods to ensure a successful C-section on the correct date (10–12). Although certain factors such as breed, age, and brachycephaly have been identified as potential risk factors for dystocia (4, 7, 13), there is no standardized and generally accepted rule on how to identify bitches that would benefit from undergoing an elective C-section (14). For this study, we used an online questionnaire distributed through breeders' clubs and social media which gave breeders of different breeds the possibility to respond to specific questions to contribute to the knowledge on potential influencing factors for dystocia and cesarean section in the dog. The aim of our study was to evaluate the incidence of C-section in a population of different breeds, to investigate contributing factors to the occurrence of C-section, and to compare data on elective and emergency C-sections (e.g., stillbirth) as well as to evaluate the impact of C-sections on the rate of stillbirth.

## Materials and methods

Data collection was performed *via* a convenience questionnaire distributed to breeders of German-speaking countries (Austria, Germany, Switzerland) as a Microsoft EXCEL® file or as an online questionnaire using the online platform Survey Monkey (https://www.surveymonkey.com) in German language. Executive committees of breeders' clubs were contacted *via* email and offered the opportunity to participate and/or distribute the questionnaire within their community of breeders. Furthermore, social media, in particular Facebook, was used to inform breeders about our research project and to distribute the questionnaire in both its original (Excel) and its online version. Data collection was taken place between October 2020 and May 2021, and no limit was set regarding the date of birth of either the bitches or the litters as long as the breeders were able to communicate the data based on the records they have kept. The collected data for bitches included breed, date of birth, number of litters, number of C-sections, and an evaluation of the breeders regarding housing, the character, the topline, and the body condition (BC) of the dam. The participants were able to classify the topline of the dam as either level, sloping, or high in the rear. The character of the bitch was classified by breeders as either (a) calm and balanced, (b) calm and playful, or (c) fearful, nervous, and/or insecure. Both factors are hardly measurable and based on the breeders impression/evaluation. The breeders were asked to insert all litters a dam had to provide a complete picture of the dam's reproductive career, regardless of the presence or absence of C-sections within the dam's history. Parity of the dam was classified as either primiparous or pluriparous. The exact number of litters in pluriparous bitches was not used in the statistical analysis. Personal information on the breeders was not collected.

The collected data for litters included date of birth, number of pups born, number of pups stillborn, and type of parturition (eutocic/cesarean section). Information collected for cases of cesarean sections included reason for C-section (emergency C-section or elective C-section), time of intervention (prior to or after the birth of the first pup), and number of pups delivered alive or dead during C-section. Parity of the bitch and the number of C-sections that have occurred prior to the date of birth of each litter were recorded. The owner was further asked to classify his experience as either unexperienced (1–2 parturitions), medium level of experience (3–4 parturitions), or high level of experience (>5 parturitions) depending on the number of births she/he had seen and followed.

Microsoft® Excel for Mac (version 16.16.25) was used to create the final dataset for statistical evaluation. All statistical analyses were performed using XLStat (Copyright Addinsoft 1995–2021) and SAS (Copyright © 2002–2022 by SAS Institute Inc., Cary, NC, USA). Descriptive statistics were calculated. Counting data (expressed as percentages) were compared using the k-proportions test, z-test for comparisons of two proportions (with correction for continuity), and chi-square test (or Fisher's exact test when required). Multiple chi-square tests were performed using the Marascuilo approach with Bonferroni's correction. For binary response variable C-section, a logistic regression was performed using the fixed effect of age (five classes) as predictors and the parity (primiparous vs multiparous), prior C-section, and the number of total pups as covariates. The effect of bitch was included in the model as a random effect (PROC GLIMMIX of SAS). Relative risk (RR) and 95% confidence intervals (95%CI) were calculated as the exponential of the estimated coefficients for the binary model. For breeds with at least 20 litters, an analysis of variance was conducted on the total number of pups using a mixed liner model with the fixed effects of breed, type of delivery (C-section vs no C-section), and interaction (PROC MIXED of SAS). The bitch effect was included in the model as a random effect. For stillborn, an ANOVA mixed model was adopted, which included the fixed effects of breed, experience of the operator, and number of prior C-sections (0,1,2) and bitch as a random effect. For all the analyses, post-hoc pairwise comparisons between least-square means were made using Bonferroni's correction.

## Results

### General aspects and information on the population

Information on a total of 423 bitches belonging to 138 breeders and their respective litters, accounting for a total of 899 litters, was collected and included in the statistical evaluation. Forty-three bitches were born between January 1, 1980, and December 31, 1999, 129 bitches were born between January 1, 2000, and December 31, 2009, and the majority of bitches (*n* = 251) were born after January 1, 2010. These bitches belonged to 80 different breeds, of which 79 were acknowledged by the Federation Cynologique Internationale (FCI), whereas one was recognized only by national kennel clubs (Silken Windsprite). The breed distribution is presented in Table 1. No bitches or litters of mixed breeds were included in our population.

**Table 1:** Breed distribution within our population.

**Breed**	**N°dams**	**N°litters**	**N°C-section**	**N°pups**	**N°stillborn pups**
American Staffordshire Terrier	2	3	1	26	0
Australian Cattle Dog	1	1	0	7	0
Australian Shepherd	6	11	0	70	2
Austrian Black and Tan Hound	1	2	1	10	0
Bearded Collie	4	12	6	87	6
Belgian Shepherd Dog	7	15	1	111	6
Berger Blanc Suisse	1	2	0	15	0
Bernese Mountain Dog	29	71	19	550	48
Black Russian Terrier	6	11	5	85	8
Border Collie	12	27	5	139	19
Border Terrier	2	5	1	16	0
Borzoi	1	1	1	2	0
Boston Terrier	1	1	1	4	1
Boxer	5	12	2	71	6
Bullmastiff	4	6	3	44	7
Bullterrier (Standard)	1	1	1	10	0
Chihuahua	2	3	0	13	3
Chow-Chow	3	5	0	24	2
Continental Bulldog	1	1	0	13	3
Continental Toy Spaniel	2	4	0	13	0
Dachshund Smooth Haired	1	1	0	5	0
Dachshund Wire-haired	18	47	14	224	4
Deerhound	4	5	0	42	0
Do Khyi (Tibetan Mastiff)	5	12	2	94	17
Doberman Pinscher	1	1	1	4	3
English Cocker Spaniel	4	10	1	48	4
English Setter	5	10	4	62	1
Entlebuch Cattle Dog	3	8	1	42	1
Eurasian	9	19	3	122	4
Flat Coated Retriever	1	2	0	11	0
French Bulldog	4	6	4	31	1
German Shepherd	10	22	2	177	19
German Wolfsspitz	1	1	0	7	0
Golden Retriever	22	49	11	353	18
Gordon Setter	10	21	1	193	8
Great Dane	3	4	2	20	4
Great Swiss	4	10	4	72	26
Groenendael	7	14	2	85	1
Hanoverian Scent Hound	2	3	0	19	7
Havanese	3	9	1	47	5
Hovawart	43	101	13	839	29
Icelandic Sheepdog	1	2	0	10	0
Irish Glenn of Imaal Terrier	3	6	4	32	0
Irish Red and White Setter	1	1	0	6	0
Irish Terrier	5	11	0	66	1
Labrador Retriever	7	12	1	110	5
Large Münsterländer	3	7	0	72	3
Manchester Terrier	1	1	0	5	0
Miniature American Shepherd	1	1	0	3	0
Miniature Bull Terrier	11	20	4	105	8
Miniature Dachshund Long-haired	4	9	1	32	1
Miniature Dachshund Wire-haired	2	3	2	6	2
Miniature Poodle	1	1	0	5	0
Miniature Schnauzer	3	7	3	27	1
Newfoundland	12	22	7	119	13
Norfolk Terrier	11	27	3	88	7
Norwich Terrier	12	31	16	99	22
Parson Russell Terrier	8	19	2	87	4
Portuguese Water Dog	1	1	0	14	0
Pug	2	4	2	12	1
Pyrenean Mountain Dog	2	3	1	32	0
Rhodesian Ridgeback	2	5	1	48	1
Rough Collie	11	15	0	87	3
Saarloos Wolfhound	1	1	0	2	1
Schipperke	4	10	2	36	1
Scottish Terrier	9	16	12	73	17
Shetland Sheepdog	8	16	4	55	3
Silken Windsprite^*^	1	1	0	7	0
Small Münsterländer	1	1	1	2	1
Smooth Collie	3	8	0	57	4
Spinone Italiano	2	4	1	34	1
Staffordshire Bullterrier	3	8	0	65	0
Terrier Brasileiro	6	7	0	52	0
Tibetan Spaniel	8	23	5	126	7
Tibetan Terrier	10	26	4	142	5
Toy Poodle	3	7	1	25	0
Welsh Corgi Cardigan	1	1	0	7	0
Welsh Corgi Pembroke	1	2	1	10	4
West Highland White Terrier	4	8	2	28	0
Whippet	3	3	1	22	0
**Total**	**423**	**899**	**194**	**5,615**	**379**

N° dams, number of bitches enrolled in the study; N° litters, number of litters included in the study; N° C-section, number of C-sections for each respective breed; N° pups, total number of pups born; N° stillborn pups, total number of stillborn pups;

* breed not recognized by the FCI.

The age of the dams at each whelping ranged between 1.1 and 10.6 years, with a mean of 4.3 ± 1.7 years. The bitches included in the sampled population had a minimum of 1 litter and a maximum of 7 litters. The mean number of litters was 2.1 ± 1.1 litters per bitch.

Information on breeders' experience at the moment of birth was available for 896 litters. Breeders described themselves as either unexperienced, medium level of experience, or high level of experience in 21, 17.6, and 61.4% of cases, respectively. No statistically significant differences were observed when evaluating the breeders' experience.

Information on housing was provided for 421 bitches. Indoor housing was defined as indoor (no access to any outdoor area such as a backyard), outdoors (housing exclusively in kennels outdoors), and mixed housing (bitches living inside the house with access to an outdoor area). The majority of bitches were kept in mixed housing (72.9%), 26.4% of bitches were housed indoors, and only 0.7% of bitches were housed exclusively outdoors.

The collected data on the character of the bitch, the BC, and the topline were as might be expected with the majority of bitches being of calm/balanced character (53.1%), normal BC (92.1%), and level topline (92%). Numbers of answers which differed from the majority were few. Therefore, these factors were not included in the statistical analysis. Information on the overall distribution of data as well as the distribution in eutocic parturitions and C-sections of breeders' experience, housing, character, BC, and topline is given in Table 2.

**Table 2:** Description of number of responses and percentages regarding breeders' experience and the dams housing, character, body condition, and topline.

		**Overall**		**Eutocic parturitions**		**C-sections**	
		***N***	**%**	***N***	**%**	**N**	**%**
Experience							
	Unexperienced	188	210%	155	22.0%	33	17.1%
	Medium level	158	17.6%	117	16.6%	41	21.2%
	High level	550	61.4%	431	61.3%	119	61.7%
	**Total**	896	**100.0%**	703	**100.0%**	193	**100.0%**
Housing							
	Only indoors	111	26.4%	72	26.6%	39	26.0%
	Indoors and outdoors	307	72.9%	196	72.3%	111	74.0%
	Only outdoors	3	0.7%	3	1.1%	0	0.0%
	**Total**	421	**100.0%**	271	**100.0%**	150	**100.0%**
Character							
	Calm/balanced	223	53.1%	139	51.3%	84	56.4%
	Calm/playful	174	41.4%	122	45.0%	52	34.9%
	Fearful/nervous/insecure	23	5.5%	10	3.7%	13	8.7%
	**Total**	420	**100.0%**	271	**100.0%**	149	**100.0%**
BC							
	Normal	385	92.1%	253	94.1%	132	88.6%
	Underweight	18	4.3%	13	4.8%	5	3.4%
	Overweight	15	3.6%	3	1.1%	12	8.1%
	**Total**	418	**100.0%**	269	**100.0%**	149	**100.0%**
Topline							
	Level	382	92.0%	249	93.3%	133	89.9%
	Sloping	10	2.4%	6	2.2%	4	2.7%
	High in the rear	23	5.5%	12	4.5%	11	7.4%
	**Total**	415	**100.0%**	267	**100.0%**	148	**100.0%**

N, number; %, percentage; Experience, breeders' experience; BC, body condition.

A total of 899 litters were included in the statistical analysis for a total of 5,615 pups. The litter size ranged between 1 and 14 pups with a mean of 6.3 ± 2.9 pups. Of all pups born, 379 were registered as stillborn with a maximum of 9 stillborn pups within one litter and a mean of 0.4 ± 0.9 pups per litter. The overall stillbirth rate was therefore 6.7%. The distribution of total number of pups and number of stillborn pups for the respective breeds is presented in Table 1.

Parity of the dam was recorded as either primiparous or pluriparous at the time of whelping. Of all litters born, 419 were born by primiparous bitches, whereas 480 were born by pluriparous bitches.

### Investigation into cesarean section

Of 899 litters, 705 (78.4%) and 194 (21.6%) litters were born *via* eutocic parturition and C-section, respectively. Of these 194 C-sections, 35 (18%) were elective procedures accounting for 3.9% of all litters born and the 159 C-sections due to parturition emergencies correspond to an incidence of 17.7% in our population. Out of the 5,615 pups born in total, 4,652 (82.8%), 116 (2.1%), and 847 (15.1%) pups were born *via* eutocic parturition, elective cesarean sections, and emergency C-section, respectively.

A size in litters born *via* an emergency C-section ranged between 1 and 14 pups with a mean of 5.3 ± 2.9 pups. A litter size in an elective C-section ranged between 1 and 9 pups with a mean of 3.3 ± 2.6 pups. Overall C-sections were significantly more common in litters with 1 or 2 pups (51.6%) than in litters with a high number of pups (>12 pups, 22.7%) or in litters with 3–11 pups (18.1%, $\chi_{11}^{2}$ = 75.81 *p* < 0.001).

When considering the timing of emergency surgery during parturition, C-section was performed in 96 litters (60.4% of emergency C-sections) prior to and in 63 litters (39.6% of emergency C-sections) after the birth of the first pup. Elective C-sections were excluded from the evaluation of this value.

**Figure 1:**
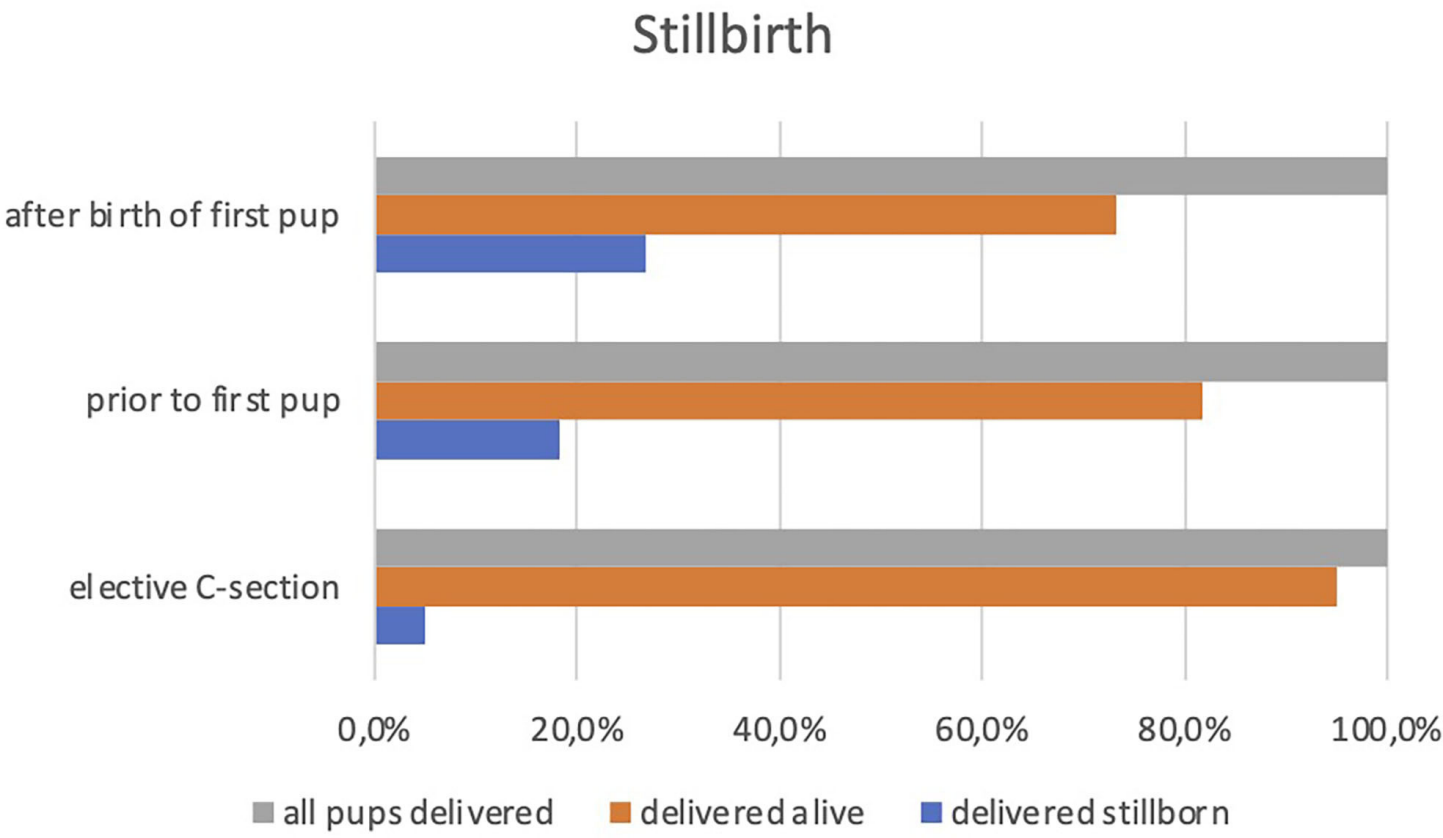
Comparison of the percentage of pups delivered alive and pups delivered stillborn during emergency C-sections performed after the birth of first pup and prior to the first pup and elective C-sections.

Of the 620 pups delivered during emergency C-sections, 20.5% were stillborn. When emergency C-sections were performed prior to the birth of the first pup, 373 pups were delivered alive, whereas 83 pups were stillborn, accounting for a rate of stillbirth of 18.2%. When emergency C-sections were performed after the birth of the first pup, 120 pups were delivered alive, whereas 44 pups were stillborn, accounting for a rate of stillbirth of 26.8%. This difference in the percentage of stillborn puppies delivered during C-sections performed prior to (18.2%) or after (26.8%) the birth of the first pup was significant (z = 2.23; *p* = 0.025). In elective C-sections, 111 pups (95.7%) were delivered alive, whereas five (4.3%) were stillborn. Mortality in elective C-sections (4.3%) was therefore significantly lower than that in emergency C-sections (z = 2.99; *p* = 0.003). Stillbirth (at least one stillborn pup) was recorded in 11.4% of all elective C-sections vs. 44.7% of emergency C-sections (z = 3.54; *p* < 0.001). A comparison of these results is shown in Figure 1. Elective C-sections were performed more frequently in litters with a single pup (12.9%) than in pregnancies with two or more pups (z = 14.51; *p* < 0.001). Of all bitches in our population, 35.5% have had at least one C-section, and prior C-sections were reported in 81 out of 899 litters (9%). Emergency C-sections were performed in 16.7% of all primiparous litters and in 18.6% of all pluriparous litters (*p* = 0.518), whereas elective C-sections were performed in 2.9 and 4.8% of all primiparous and pluriparous litters, respectively. Of all emergency C-sections, 56% have been performed in pluriparous litters. A similar result has been found for elective C-sections as the majority of them (65.7%) were performed in pluriparous litters. In order to further assess the role of age on the incidence of cesarean sections, we divided the bitches into the following five age groups: group 1 < 2 years, group 2 between 2 and 4 years, group 3 between 4 and 6 years, group 4 between 6 and 8 years, and group 5 > 8 years. The observable increase in the incidence of cesarean section with increasing age was not statistically significant ($\chi_{4}^{2}$ = 6.29; *p* = 0.172). However, when considering parity of the dam within each age group, there was a significant increase in the incidence of cesarean sections in primiparous bitches with increasing age (12.2, 17.2, 27, and 50%, respectively, in groups 1–4; $\chi_{3}^{2}$ = 13.32 *p* = 0.004). The logistic regression confirmed that age and parity were not significant regarding the C-section when evaluated separately, whereas prior C-section and total number of pups were significant. The presence of prior C-section increased the risk of a C-section in the following whelpings 4-fold (RR = 4.54; 95%CI 2.56–7.70; *p* < 0.001). Regarding litter size, the logistic regression has shown a decrease for the risk of C-section for each pup more up to 11 pups (RR = 0.80; 95% CI 0.75–0.86; *p* < 0.001). The results of the logistic regression remained the same after the exclusion of elective C-sections from the evaluation.

### Breed-specific evaluation

To provide a breed-specific evaluation, we included only breeds that were represented with at least 20 litters as given in Tables 3, 4. A significant difference between these breeds was observed in the overall incidence of C-sections ($\chi_{12}^{2}$ = 38.46; *p* < 0.001), with the Norwich Terrier having the highest (51.6%) and the Gordon Setter having the lowest (4.8%) incidence of C-section. The Norwich Terrier was also the only breed in which C-sections in age group 1 (< 2 years) have been reported. Bernese Mountain Dogs and Newfoundland bitches had a greater litter size when experiencing eutocic parturitions compared with litters born *via* C-sections (*p* < 0.001). The mean litter size in Bernese Mountain Dogs was 8.5 ± 2.5 pups in eutocic parturitions and 5.6 ± 3 pups in litters born *via* C-section. In the Newfoundland, the mean litter size in eutocic parturitions was 6.8 ± 3.2 pups and 2.4 ± 1.5 pups in litters born *via* C-section. Information on the litter size based on the type of parturition for each breed is given in Table 4. We furthermore observed a significant difference in the number of stillborn pups among breeds (*p* < 0.001), with the Norwich Terrier having the highest (22.2%) and the wire-haired Dachshund the lowest (1.8%) stillbirth rate. When evaluating the incidence of stillbirth in C-sections, we may say that the German Shepherd (71.4%) and the Border Collie (50%) both had a significantly higher number of stillborn pups compared with the other breeds (11.8%, *p* < 0.001). Potential differences between emergency C-section and elective C-section could not be evaluated in a breed-specific manner due to the limited amount of data per breed.

**Table 3:** Breed-specific evaluation.

**Breed**	**Bitches**	**Litters**	**CS**	**CS (%)**	**ECS**	**Total pups**	**Pups stillborn**	**Stillbirth (%)**
Bernese Mountain Dog	29	71	19	26.8	3	550	48	8.7
Border Collie	12	27	5	18.5	0	139	19	13.7
Dachshund Wire-Haired	18	47	14	29.8	3	224	4	1.8
German Shepherd	10	22	2	9.1	0	177	19	10.7
Golden Retriever	22	49	11	22.4	3	353	18	5.1
Gordon Setter	10	21	1	4.8	0	193	8	4.1
Hovawart	43	101	10	9.9	0	839	29	3.5
Miniature Bullterrier	11	20	6	30	1	105	8	7.6
Newfoundland	12	22	7	31.8	1	119	13	10.9
Norfolk Terrier	11	27	3	11.1	1	88	7	8.0
Norwich Terrier	12	31	16	51.6	2	99	22	22.2
Tibetan Spaniel	8	23	6	26.1	0	126	7	5.6
Tibetan Terrier	10	26	4	15.4	0	142	5	3.5

CS, number of C-sections; CS (%), incidence of C-section in percent; ECS, number of elective C-section total pups, number of all pups born; pups stillborn, number of stillborn pups; Stillbirth rate in % without consideration of type of parturition.

**Table 4:** Breed-specific evaluation of litter sizes depending on type of parturition; litter size is given in mean ± SD of pups per litter.

**Breed**	**C-section (mean ±SD)**	**Eutocic parturition** ** (mean ±SD)**	**Overall litter size** **(mean ±SD)**
Bernese Mountain Dog	5.6 ± 3.0	8.5 ± 2.5	7.7 ± 2.9
Border Collie	4.8 ± 2.0	5.2 ± 1.2	5.1 ±1.3
Dachshund Wire-Haired	4.3 ± 1.4	5.0 ± 1.6	4.8 ± 1.5
German Shepherd	7.0 ± 0.0	8.2 ± 2.5	8.0 ± 2.4
Golden Retriever	5.3 ± 3.6	7.8 ± 2.1	7.2 ± 2.7
Gordon Setter	14.0	9.0 ± 3.5	9.2 ± 3.6
Hovawart	7.6 ± 3.3	8.4 ± 2.2	8.3 ± 2.3
Miniature Bullterrier	4.7 ± 1.9	5.5 ± 1.9	5.3 ± 1.9
Newfoundland	2.4 ± 1.5	6.8 ± 3.2	5.4 ± 3.5
Norfolk Terrier	2.3 ± 0.6	3.4 ± 1.3	3.3 ± 1.3
Norwich Terrier	2.8 ± 1.1	3.6 ± 1.5	3.2 ± 1.3
Tibetan Spaniel	5.0 ± 1.3	5.6 ± 1.6	5.5 ± 1.5
Tibetan Terrier	5.0 ± 1.4	5.5 ± 1.4	5.5 ± 1.4

C-section, litter size in litters born *via* C-section; eutocic parturition, litter size in litters born in eutocic parturition; overall litter size, litter size for each breed regardless of type of parturition.

## Discussion

Dystocia in the canine species is a frequently encountered emergency (14, 15). Although pharmacological treatments are available, the majority of bitches presenting with dystocia need to undergo a cesarean section (1, 6, 13). Several risk factors for the occurrence of dystocia have been described in various publications (1, 4, 8, 13, 15, 16), including breed, litter size, size of the fetus in regard to the dimension of the pelvic canal, and others. C-section has long been considered an emergency surgery (8, 17); however, the increasing reports on the benefits of elective C-sections have led to the conclusion that in certain cases an elective C-section, regardless of anesthesiological and surgical risks, has more benefits for both the mother and the litter than disadvantages (1, 9, 14). It has been reported that especially breeders of breeds highly at risk opt more frequently for an elective C-section (4, 18). Therefore, different protocols have been described regarding the timing and preparation for elective C-sections as the correct timing is crucial to the survival of the neonates (10–12, 16, 19). Although little information may be found in the literature regarding the incidence of elective C-sections in populations composed of different breeds, an incidence of up to 32% has been reported (14). In populations of breeds at particularly high risk of dystocia, such as the English Bulldog (20), the incidence of elective C-sections may even be approaching 100% (21). Within our population, the incidence of elective C-sections was much lower, accounting for 3.9% of all parturitions. An emergency C-section on the contrary was performed in 17.7% of litters within our population, which is slightly higher than the incidence of 16% reported by Bergström et al. (13) and much higher than the incidence of around 5% reported earlier by Linde-Forsberg (22). Such a difference may be due to the type of data collection as breeders may be more prone to participate in studies like the present if they encountered frequent dystocia and if emergency C-sections are common within their own kennel or breed. Interestingly, brachycephalic breeds were underrepresented within our population contrary to what has previously been reported (7). This lack of representation of brachycephalic breeds within our results does not imply a low risk of emergency C-sections within these breeds, but rather a consequence of type of data collection and a lack of participation of breeders of brachycephalic breeds. The bitches in our study C-sections were performed more frequently in small litters (1 or 2 pups) and large litters of above 12 pups, consistent with the previously reported results (15).

Although emergency and elective C-sections were performed in both primiparous and pluriparous bitches of our studied population, the majority of both elective and emergency C-sections were performed in pluriparous bitches. This result is in disagreement with that of previous reports of a higher risk of dystocia in primiparous bitches (23, 24). One possible explanation is that whenever a bitch may have had problems at her first parturition, the breeders may have decided to request an elective procedure also for the subsequent litter/litters. However, the number of elective C-sections in our study is too low to allow trustworthy conclusions in this regard. Further research with a larger population is needed to better understand and evaluate the motivations breeders might have to request elective C-sections. Furthermore, it is possible that a certain number of bitches within our population experienced dystocia during their first pregnancy which was then resolved without the use of emergency C-section. Further investigation is needed to better understand the incidence of dystocia without the subsequent emergency C-section.

Age has an important impact on pregnancy and delivery in the human and is considered a risk factor for problems during pregnancy as well as for the occurrence of dystocia. Especially primiparous women with an age of >35 years are generally considered as having a geriatric pregnancy with a concomitant increase in risks (25, 26). When age was evaluated within our canine population, the increase in the incidence of C-sections with increasing age was observable, but not statistically significant (*p* = 0.172). Yet, once parity was taken into consideration, we were able to see a statistically significant impact of increasing age in primiparous bitches, leading to a higher incidence emergency C-section (*p* = 0.004). The relationship between age of the dam and incidence of C-sections has previously been investigated with contradicting results (23, 27). The observation of increasing incidence of C-section with increasing age in our population is in disagreement with what has been described previously based on a population composed of different breeds, in which the highest incidence was found within the group of bitches aged between 3 and 5.9 years (4), compared with the highest incidence in bitches aged >8 years in our population. On the contrary, Linde-Forsberg and Persson (27), Bergström et al. (28), and Cornelius et al. (15) are in agreement with our results of increasing incidence of C-sections with increasing age at parturition, regardless of the parity. Münnich and Küchenmeister (1) reported an increased risk of dystocia for singleton pregnancies, which may be characterized by uterine disorders and prolonged parturition in older bitches. Both age and parity are widely known as risk factors for dystocia and subsequent necessary C-section. However, the impact of advanced age at first parturition on the risk of C-section is not described in the recent literature, yet breeders may consider it as an important factor that influences their decision on whether or not to breed and/or perform an elective C-section. Within this study, parity of the bitch was considered as either primiparous or pluriparous without consideration of the exact number of litters born by each bitch. Although parity has shown its importance when combined with the effect of age also in this binary method of classification, further evaluations will be needed to understand whether such an effect is also influenced by the number of litters a bitch may have had.

Data on history of prior C-sections for the bitches in our study show that a prior C-section increases the risk of the need of C-sections in the subsequent whelpings 4-fold. Despite the high number of studies on risk factors for dystocia in the canine, the role of prior C-sections as a risk factor is rarely reported (9, 23). In a work by Proctor et al. (18), 25 of 149 bitches had a history of prior C-section and nearly half of these bitches underwent an elective C-section at the end of the following pregnancy. Another very important factor driving the decisions of many breeders toward an elective C-section is the fear of stillbirth. As previously mentioned, stillbirth has a high incidence in the canine species, and our results have confirmed that stillbirth is significantly lower in elective C-sections compared with that in emergency C-sections as has been previously described (6, 15). Moreover, the number of litters that did not have any stillborn pups was significantly lower in elective C-sections. Although overall mortality was higher in emergency C-sections, timing was of significant importance. The rate of stillbirth was higher in emergency C-sections performed after the birth of the first pup compared with C-sections performed prior to the birth of the first pup. The incidence of stillbirth within our population of litters undergoing an emergency C-section is slightly lower (if performed prior to the birth of the first pup) or slightly higher (if performed after the birth of the first pup) compared with that of the literature (29). This result may be explained by the fact that C-sections prior to the expulsion of the first pup are usually performed much earlier after the onset of parturition and prolonged birth has been defined as a possible risk factor for stillbirth (1, 15, 30). Our breed-specific evaluation includes breeds that are represented with at least 20 litters. Similar to other publications (13), we did not encounter breeds that have been previously described as being at high risk of dystocia with subsequent C-section with a number of litters above this threshold. On the contrary, some breeds represented with a rather high number of litters such as the Hovawart are rarely mentioned in the literature. On the one hand, the breed with the highest incidence of cesarean section within our population was the Norwich Terrier, which was also the only breed with C-sections recorded in the age group under 2 years. On the other hand, the Gordon Setter had the lowest incidence of C-sections of all included breeds. Such a difference between the Norwich Terrier and the Gordon Setter can be observed also in the study of Evans et al. (20). Eleven of the 13 breeds included in our study were represented in the study of Evans et al. (20). The reported incidences of cesarean section are similar in the Dachshund yet differ in some cases greatly such as in the Miniature Bullterrier and the Norwich Terrier compared with the results of our study. This may be due to the much lower number of litters in our study, as well as due to the difference in questionnaire distribution between the two studies. Although the included breeds have rarely been mentioned in previous studies as at-risk breeds (7, 13), differences in the incidence of C-section between breeds were statistically significant (*p* < 0.001). Although a litter size has been mentioned as a risk factor in the evaluation of the general population, the impact is especially evident in the Bernese Mountain Dog and the Newfoundland. The Norwich Terrier had not only the highest incidence of C-section but also the highest incidence of stillbirth (22.2%) compared with other breeds such as the wire-haired Dachshund that had the lowest incidence of stillbirth with 1.8%. Considering that an emergency C-section has been reported to have an increased rate of stillbirth and that the Norwich Terrier had the highest incidence of C-section, the results may not be reported separately, as the high incidence of emergency C-section may be either cause or consequence of the high rate of stillbirth. Based on the data we collected, a distinction between cause and consequence in the case of the Norwich Terrier is not possible. Although the Norwich Terrier has the highest overall stillbirth rate, the German Shepherd and the Border Collie show a surprisingly high rate of stillbirth in C-sections. Yet, it has to be considered that in both breeds all C-sections were emergency C-sections and that particularly in the German Shepherd the percentage reported is based on two C-sections, which resulted in the birth of 14 pups of which 10 were stillborn. We consider such a result hardly representative for the breed and rather an exception, which may be due to health problems of the dam, the litter, or both. In the case of the Border Collie instead, the number of C-sections is still low with five C-sections due to dystocia accounting for a total of 24 pups of which 12 were stillborn. Further investigation with a higher number of litters and C-sections is needed for these two breeds to evaluate the importance of our results. Furthermore, breeds that were not included in the breed-specific analysis (e.g., Scottish Terrier) had relatively high numbers of emergency C-sections, yet due to their overall low number of bitches and litters, no conclusions may be drawn in this regard (Table 1).

Evaluation on housing, character, BC, and topline of the dam has been merely descriptive, whereas breeders' experience showed no statistical significance. Further data collection with more detailed questions will be needed to increase the number of bitches in order to evaluate the importance of housing, character, BC, and topline on the occurrence of emergency C-section.

All information on our population was collected using a questionnaire without inclusion criteria. This means that breeders were able to participate regardless of the breed they are breeding, the experience they have, or other factors. This fact allowed us to obtain a large number of litters from bitches of 80 different breeds which differ not only in phenotypical appearance but also in other anatomical and behavioral peculiarities. We furthermore included in the general evaluation all breeds to evaluate the overall incidence of C-section in a canine population as well as other factors such as rate of stillbirth. Although the lack of limitations for participation may be considered an advantage as we were therefore able to enroll also lesser-known breeds, it has also some disadvantages. First of all, it has to be considered that owners and breeders of certain breeds may have been more interested in investing in and in contact with the topic of dystocia and C-section than others, which made them more prone to contribute. This may be due to the fact that their breed has a higher incidence of dystocia, although we do not consider this as the main reason, as few breeders of known at-risk breeds responded to the questionnaire. It may also be due to the fact that the club of these breeders are particularly aware of the risks and consequences of dystocia and motivate their breeders more to contribute to studies such as ours. We have seen such an increased interest and motivation by the breeders' club in particular in the Hovawart breed which contributed a total of 101 litters to this study. It is a known fact that information obtained by questionnaires has to be considered with a certain caution as none of the reported information has been verified by a veterinary professional in the moment of birth. Yet, the contribution was completely voluntary, and no reward was granted for participation. All breeders included in this study are from German-speaking countries (Germany, Switzerland, Austria). Although policies vary between clubs, it is common practice within breeders' clubs of these countries to obligate the breeder to provide detailed information on the mating, the birth, and the litter itself, which is also controlled by the club itself. Therefore, it is of interest to the breeder to provide and keep clean and detailed records. We therefore consider the information collected and the population evaluated as an overall good trustworthy and representation of the reality.

## Conclusion

Our study provided an insight into possibly influencing factors on the occurrence of C-section, its overall incidence, and information on the rate of stillbirth in emergency and elective C-sections in the general population. Furthermore, less frequently studied breeds such as the Hovawart and the Norwich Terrier have been investigated in a breed-specific manner, which makes our contribution valuable. An increase in the number of dams and number of litters will be necessary to provide further insight into an emergency C-section in these breeds. The results of this study further indicate an important influence of age of primiparous bitches on the incidence of C-sections. The rate of stillbirth was significantly higher in emergency C-sections when compared with that in elective C-sections. Furthermore, the timing of intervention in the cases of emergency C-sections had an impact on the rate of stillbirth within our population. We therefore conclude that elective C-section in primiparous bitches of advanced age and close monitoring of the parturition particularly in the early stages may therefore be advised to allow fast intervention in the case of dystocia and to assure an increase in puppy survival. A similar conclusion can be drawn regarding bitches that have already undergone C-section in prior parturitions as the risk of the necessity of emergency C-section in successive whelpings is increased.

## Data availability statement

The raw data supporting the conclusions of this article will be made available by the authors, without undue reservation.

## Ethics statement

Ethical review and approval was not required for the animal study because the study is based on a collection of retrospective data without the necessity of performing any type of experiments or actions on the animals.

## Author contributions

MS and AM were involved in conceptualization and writing—review and editing. MS and BC were involved in methodology and data curation. BC was involved in formal analysis. MS was involved in investigation and writing—original draft preparation. AM was involved in supervision. All authors have read and agreed to the published version of the manuscript.

## Conflict of interest

The authors declare that the research was conducted in the absence of any commercial or financial relationships that could be construed as a potential conflict of interest.

## Publisher's note

All claims expressed in this article are solely those of the authors and do not necessarily represent those of their affiliated organizations, or those of the publisher, the editors and the reviewers. Any product that may be evaluated in this article, or claim that may be made by its manufacturer, is not guaranteed or endorsed by the publisher.
